# A patient with extensive Darier disease efficiently treated and maintained on doxycycline monotherapy

**DOI:** 10.1016/j.jdcr.2025.01.006

**Published:** 2025-01-31

**Authors:** Avik Mondal, Peeyush Kiran Tripathy, Tarun Sharma

**Affiliations:** Department of Dermatology & Venereology, AIIMS, Kalyani, India

**Keywords:** Darier disease, doxycycline, dyskeratosis, genodermatosis, treatment

## Introduction

Darier disease (DD) occurs due to a gene mutation of ATP2A2 on chromosome 12 and has a protracted course marked by flareups and remissions.^1^^,^^2^ Clinical features include chronic eruption of greasy hyperkeratotic papules and plaques, which typically occur over seborrheic areas and specific nail changes. Additionally, impacted are mucous membranes. The management of this genetic disease might provide obstacles and frequently disappointing.^3^ Segmental DD is another uncommon variant that can manifest as unilateral lesions along the Blaschko lines and is believed to be heritable exclusively in cases when mosaicism involves the gonads and is induced by a postzygotic somatic mutation.^4^ The management involves various topical and systemic options aimed at controlling symptoms and improving quality of life.^1^^,^^5^

## Case presentation

**Fig 1:**
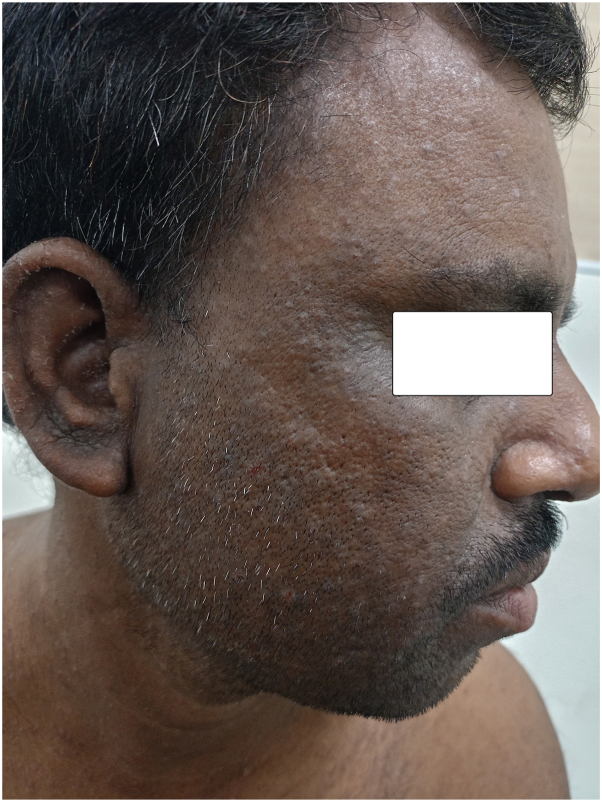
Picture showing multiple discrete greasy skin-colored to hyperpigmented papules with some excoriation over the right side of the face.

**Fig 2:**
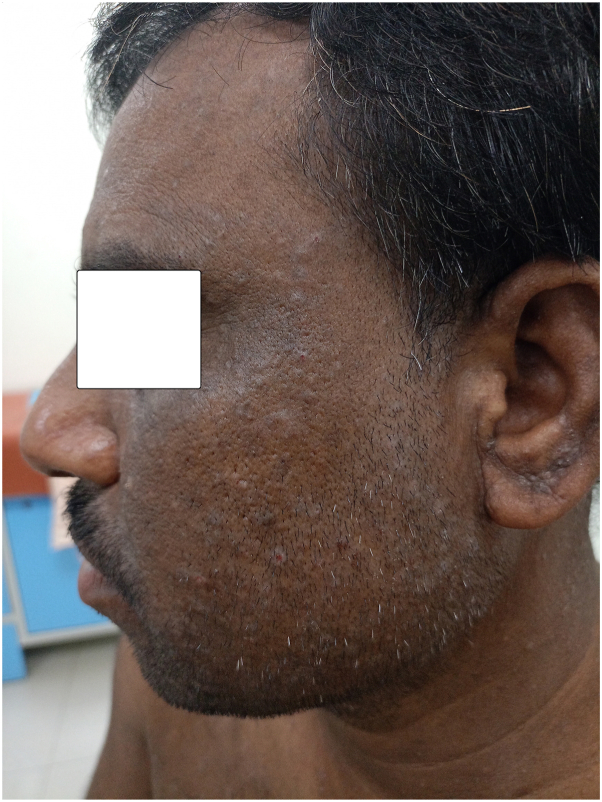
Showing multiple discrete greasy skin-colored to hyperpigmented variably excoriated papules over the left side of the face.

A 41-year-old farmer presented with multiple skin-colored to hyperpigmented malodorous keratotic greasy papules with fine white scaling along with generalized dryness distributed over the face (prominently over beard area, side of the face), neck, anterior aspect of the chest, and upper back in a seborrheic distribution (Figs 1 and 2). He has been suffering from this condition since his childhood with a history of exacerbation in summer. He did not provide any history of similar disease in his family members. Previously, he did apply mometasone furoate 0.1% lotion, prescribed by some nondermatologists. However, no improvement was observed. He never underwent any biopsy.

**Fig 3:**
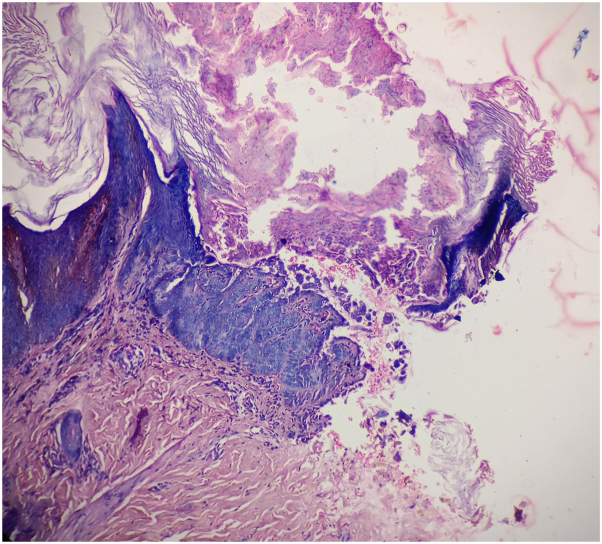
Biopsy from the papules from the chest showed suprabasilar cleft with acantholysis and dyskeratotic cells, called corps ronds in the granular layer and grains in the stratum corneum (H&E stain, original magnification: ×100). *H&E*, Hematoxylin and eosin.

**Fig 4:**
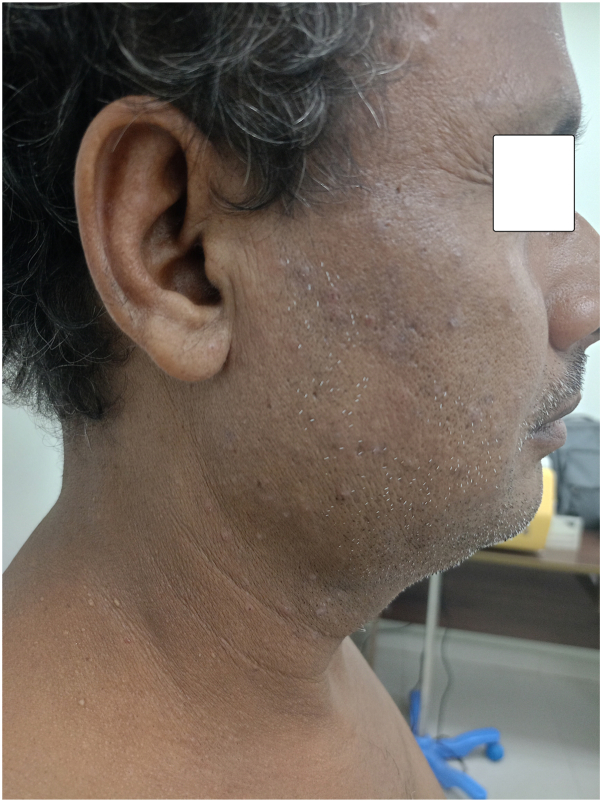
Showing complete improvement of lesions over the right side of the face.

**Fig 5:**
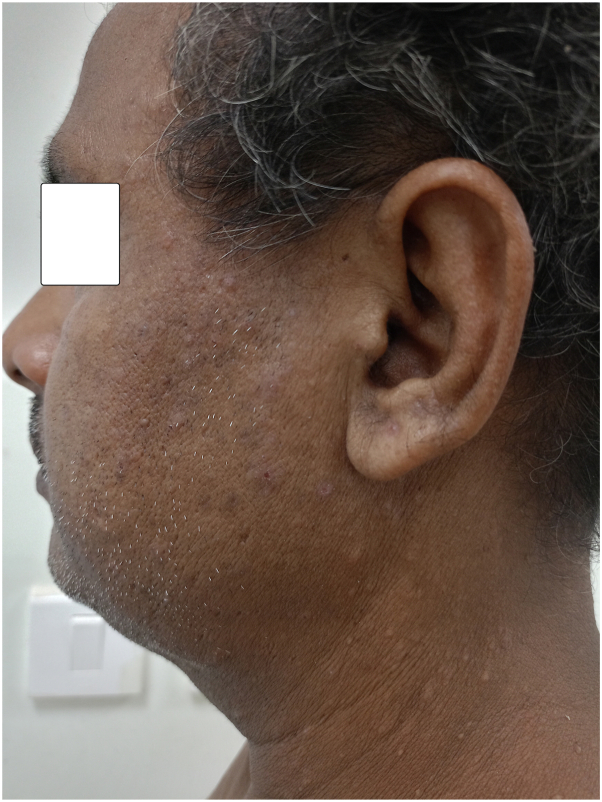
Showing almost complete resolution of lesions over the left side of the face.

After mucocutaneous examination, we found there was no involvement of the mucosa and nails. A 4-mm punch biopsy from a keratotic papule over the upper chest revealed suprabasilar acantholytic dyskeratotic cells, corps ronds, and grains, suggestive of DD (Fig 3). As the patient already applied topical corticosteroid for several instances with failure, in view of extensive involvement, we decided to put him on doxycycline 100 mg twice daily for 1 month based on previous 2 case reports and reviewed him after 1 month. He took doxycycline 100 mg twice a day and improved after 1 month (Figs 4 and 5). The dose was reduced to once a day and he was well maintained on this dose with significant control of the DD and no adverse effect was found on further follow ups.

## Discussion

The calcium pump known as sarcoplasmic/endoplasmic reticulum calcium ATPase 2 (SERCA2) is encoded by the ATP2A2 gene, which is mutated in DD.^5^^,^^6^ In order to keep the level of cytoplasmic calcium low, SERCA2 aggressively moves calcium ions from the cytosol into the endoplasmic reticulum’s lumen. This process is crucial for maintaining cellular calcium homeostasis. It has been demonstrated that blocking the SERCA pump reduces endoplasmic reticulum calcium reserves and impairs intercellular adhesion. The molecular pathways by which particular ATP2A2 mutations modify the SERCA2 protein’s activity and result in histological features such as dyskeratosis and acantholysis.^7^^,^^8^

In terms of treatment for extensive DD, oral retinoids such as acitretin or isotretinoin are the cornerstone of the treatment. However, alopecia, mucosal dryness, skin fragility, increased triglycerides, transaminitis, and impaired renal function are some serious adverse effects of oral retinoid therapy, despite being a very effective treatment for DD.^1^^,^^5^^,^^6^ Doxycycline functions as an ionophore by chelating calcium ions and promoting their passage through cell membranes. Thus, reestablishing cell-to-cell adhesion by restoring intracellular and endoplasmic reticulum calcium reserves which is impaired in DD.^5^^,^^6^ Additionally, they inhibit matrix metalloproteinases, demonstrating anticollagenase activity which has been demonstrated to contribute to the etiology of DD.^6^ These aforementioned mechanisms are responsible for the improvement seen with doxycycline in DD.

## Conclusion

To our knowledge, this is the third case report that shows significant improvement with doxycycline which provides a cost effective and safe treatment as well as a maintenance option for a recalcitrant disease such as DD with almost no long-term side effects.

## Conflicts of interest

None disclosed.

## References

[1] Cooper S.M., Burge S.M. (2003). Darier's disease: epidemiology, pathophysiology, and management. Am J Clin Dermatol.

[2] Sakuntabhai A., Ruiz-Perez V., Carter S. (1999). Mutations in *ATP2A2*, encoding a Ca^2+^ pump, cause Darier disease. Nat Genet.

[3] Sehgal V.N., Srivastava G. (2005). Darier's (Darier-White) disease/keratosis follicularis. Int J Dermatol.

[4] Barfield R.L., Barrett K.R., Moon C.M., David-Bajar K. (2002). Pruritic linear papules on a 75-year-old woman: a case of localized Darier-White disease. Cutis.

[5] Sfecci A., Orion C., Darrieux L., Tisseau L., Safa G. (2015). Extensive Darier disease successfully treated with doxycycline monotherapy. Case Rep Dermatol.

[6] Pettit C., Ulman C.A., Spohn G., Kaffenberger J. (2018). A case of segmental Darier disease treated with doxycycline monotherapy. Dermatol Online J.

[7] Foggia L., Aronchik I., Aberg K., Brown B., Hovnanian A., Mauro T.M. (2006). Activity of hSPCA1 Golgi Ca^2+^ pump is essential for Ca^2+^-mediated Ca^2+^ response and cell viability in Darier disease. J Cell Sci.

[8] Lytton J., Westlin M., Hanley M.R. (1991). Thapsigargin inhibits the sarcoplasmic or endoplasmic reticulum Ca-ATPase family of calcium pumps. J Biol Chem.

